# Space and Control in Soccer

**DOI:** 10.3389/fspor.2021.676179

**Published:** 2021-07-16

**Authors:** Florian Martens, Uwe Dick, Ulf Brefeld

**Affiliations:** Machine Learning Group, Leuphana University of Lüneburg, Lüneburg, Germany

**Keywords:** soccer (football), movement model, motion model, pitch control, soccer analytics

## Abstract

In many team sports, the ability to control and generate space in dangerous areas on the pitch is crucial for the success of a team. This holds, in particular, for soccer. In this study, we revisit ideas from Fernandez and Bornn (2018) who introduced interesting space-related quantities including pitch control (PC) and pitch value. We identify influence of the player on the pitch with the movements of the player and turn their concepts into data-driven quantities that give rise to a variety of different applications. Furthermore, we devise a novel space generation measure to visualize the strategies of the team and player. We provide empirical evidence for the usefulness of our contribution and showcase our approach in the context of game analyses.

## 1. Introduction

An important aspect when analyzing soccer games is how much space on the soccer pitch is *controlled* by teams and players at any point during a game. While, in general, control is a rather flexible term in soccer and includes the ability of a player to control the ball or the ability of a team to control possession, we focus on spatial control, that is, control of areas on the pitch. This concept has been introduced by Taki et al. (1996) who developed the concept of a *dominant region* of a player that defines the area on the pitch that is controlled by that player. That is, a player is expected to reach any point in her dominant region before any other player. These regions are derived from the so-called *motion* or *movement models* that are able to predict whether a player can reach a certain point on the pitch in a given time.

Dominant regions have the advantage that they can be visualized by partitioning a soccer pitch into areas around players that they have control over and can thereby be easily interpreted. Interpretability is a key factor to empower non-technical staff, such as coaches or game analysts, to understand data-driven results and turn them into actionable insights. Therefore, dominant regions have been frequently used as the basis for research questions on higher-levels, such as the evaluation of passes or spatial pressure (Taki and Hasegawa, 2000; Gudmundsson and Wolle, 2014; Ueda et al., 2014; Horton et al., 2017; Brefeld et al., 2019). In this line of work, Fernandez and Bornn (2018) understand control on the pitch as a continuous spatial quantity. That is, instead of assigning every point on the pitch to exactly one team, they compute a value that measures how much control a team has over a position. Their concepts are intuitive and interpretable but suffer from a too coarse player influence model. Our first contribution is to remedy this limitation by incorporating data-driven movement models as the underlying motion model (Brefeld et al., 2019). Secondly, we provide empirical results showing that the data-driven approach leads to realistic measurements of space. Thirdly, we propose new metrics for passers and pass receivers on the basis of data-driven quantification of space.

Empirical results are computed on positional data from 54 Bundesliga games from season 2017/18. We show that identifying the influence with movements of the player leads to high correlations with quantifiable outcomes such as shots on target, expected goals, and the market value of players. Finally, we showcase the benefit of the usefulness of our approach on the example of opponent analysis.

The remainder is structured as follows. Section 2 reviews related work, and section 3 introduces basic player influence models. Section 4 details our approaches to quantify space, and section 5 presents a novel space generation metric together with empirical findings. Section 6 concludes.

## 2. Related Work

Dominant regions are studied in many publications. A general definition refers to dominant regions of a player as the region on a pitch which can be reached by this very player before any other one (Gudmundsson and Horton, 2017). Taki et al. (1996) first introduced this concept based on a simple motion model that incorporates the acceleration and direction of a player. Their approach constitutes a significant improvement to simple Voronoi region models (Taki et al., 1996; Taki and Hasegawa, 2000), which simply credit space to the closest available player, ignoring running direction, or speed. Further improvements to this basic model are presented by Fujimura and Sugihara (2005) who include a resistive force to bound the, otherwise infinite, acceleration as in Taki et al. (1996). By contrast, Brefeld et al. (2019) introduce a purely data-driven probabilistic movement model using sampled trajectories of each individual player. The model can be used to derive densities of locations of player and convex hulls for all reachable points on the pitch for a predefined time window that again can be translated to dominant regions.

The previously mentioned approaches treat control as a binary variable such that every location is either controlled by one or the other team. Fernandez and Bornn (2018) also rate controlled areas on the field but propose a *continuous* measure of control that is based on the influence of each player on a given point on the pitch at a given time. They use a general Gaussian influence model, in which the covariance matrix of each bi-variate Gaussian is defined by the velocity vector of a player's and her distance to the ball. Further, the authors value space on the pitch itself. Clearly, occupied zones that are close to the goal of the opponent are of higher value than open and unoccupied space in the center of the pitch (Link et al., 2016). The authors rate areas that are usually controlled by defensive players given a certain location of the ball. They use this concept to measure how well players are able to occupy and gain space during a game. In fact, they empirically show, albeit using only data from a single game, that top players such as Lionel Messi or Andres Iniesta are able to actively occupy higher valued space than others (Fernandez and Bornn, 2018). However, the analysis does not involve movement models or movement characteristics of an individual player; individual differences such as maximum speed, acceleration, and agility are ignored. Similarly to the approaches mentioned above, the proposed model is not quantitatively evaluated.

Dominant regions are used to analyze different aspects of soccer. Some studies use dominant regions to evaluate passes. Taki and Hasegawa (2000) and Nakanishi et al. (2010) estimate the success of a pass along a straight line by measuring whether it ends in the dominant region of the receiver. Horton et al. (2017) estimate the quality of a pass by using a prediction model that, among other features, uses features based on dominant regions to learn a human rating of observed passes. Some of those features also use a measure of defensive pressure that, based on dominant regions, estimate whether defending players are able to put the passing player under enough spatial pressure to influence the outcome of the pass. A similar concept was used in Taki and Hasegawa (2000) who also measure spatial pressure based on dominant regions. Ueda et al. (2014) analyze defensive and offensive positioning depending on the location where the ball was acquired. For pitch control (PC) introduced by Fernandez and Bornn (2018), however, such evaluations are missing so far.

Several other approaches that model the movements of the player exist, however. Recently, models that make use of reinforcement learning and deep learning techniques led to impressive results such as the study of Le et al. (2017) on deep imitation learning, who show that the movements of the player can be predicted over time periods up to several seconds. Dick and Brefeld (2019) use reinforcement learning in combination with deep convolution networks to predict the dangerousness of an offensive situation. Their model is purely data-driven and works without any expert or prior knowledge. The drawback of such methods, however, is their lack of interpretability that makes it hard for experts to take actions on these insights. This is an issue that, for example, Mortensen and Bornn (2019) attempt to tackle by modeling the movements of the player in basketball with Markov transitions as Poisson point processes.

Other studies are based on similar ideas. For basketball, Franks et al. (2015) take a similar approach to rate shots based on spatiotemporal features of defending players. Link et al. (2016) also include distances of the players to the goal to quantify the dangerousness of offensive actions, and Hobbs et al. (2018) use the notion of defensive disruption as a measure of how far defenders deviate from their preferred positions in similar situations and compute transition values for the offensive team.

## 3. Influence of Player on the Pitch

### 3.1. Data

The data that are used in this study are provided by a European top-flight soccer league. The data include 54 Bundesliga games from season 2017/18. The data stems from two main sources: (i) tracking the player and ball position and (ii) event data. The former is automatically captured from video footage at 25 frames per second by the data provider. At each frame, the (*x, y*) coordinates of all 22 players plus the ball are listed. The event data consist of manually recorded in-game events such as passes, shots, and tacklings etc. Such events are collected by human observers who tag each event and enrich them with additional, event-specific information such as passing player, pass receiver, and shot success. For both data sources, (*x, y*) coordinates relate to a pitch size of 105×68 m. The center of the pitch is always at the origin (0, 0), and positions are scaled to a [−52.5, 52.5] range on the x-axis and to a [−34, 34] range on the y-axis. The timestamps of the two data sets need to be aligned so that instances from both sources can be processed together.

### 3.2. Gaussian Influence Models

An analysis of space and control requires a model of a of the influence of a player on the current situation of the game, that is, the spatial and temporal configuration on the pitch. Fernandez and Bornn (2018) model the influence of a player by a bivariate normal distribution to quantify the amount of control at a position **p** ∈ ℝ^2^ for a player *i* at position ${\text{p}}_{t}^{i}$ and time *t*,

$$\begin{array}{l} f_{t}^{i}\left(\text{p}\right)=\frac{1}{\sqrt{{\left(2\pi \right)}^{2}|\Sigma_{t}^{i}|}}\exp\left(-\frac{1}{2}{\left(\text{p}-\mu_{t}^{i}\right)}^{T}{\left(\Sigma_{t}^{i}\right)}^{-1}\left(\text{p}-\mu_{t}^{i}\right)\right). \end{array}$$

**Figure 1:**
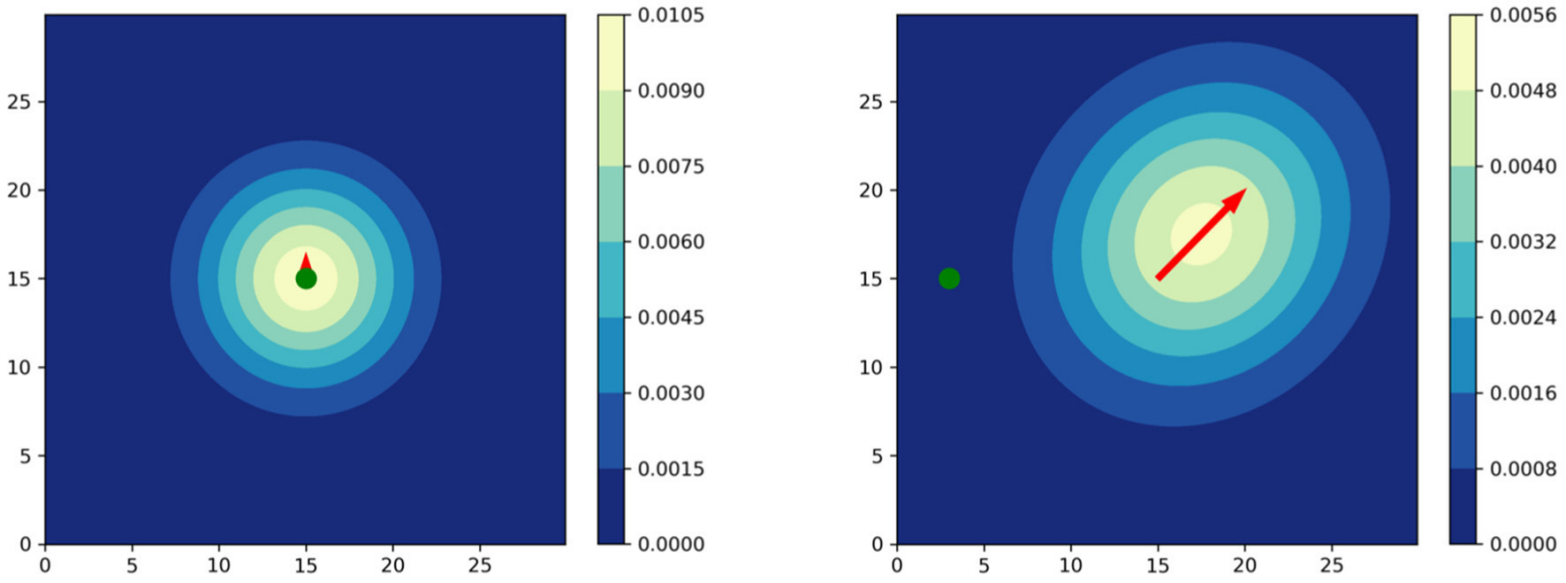
Player influence models according to Fernandez and Bornn (2018). The ball is visualized in green. **(Left)** Player standing with the ball. **(Right)** Player moving away from the ball.

**Figure 2:**
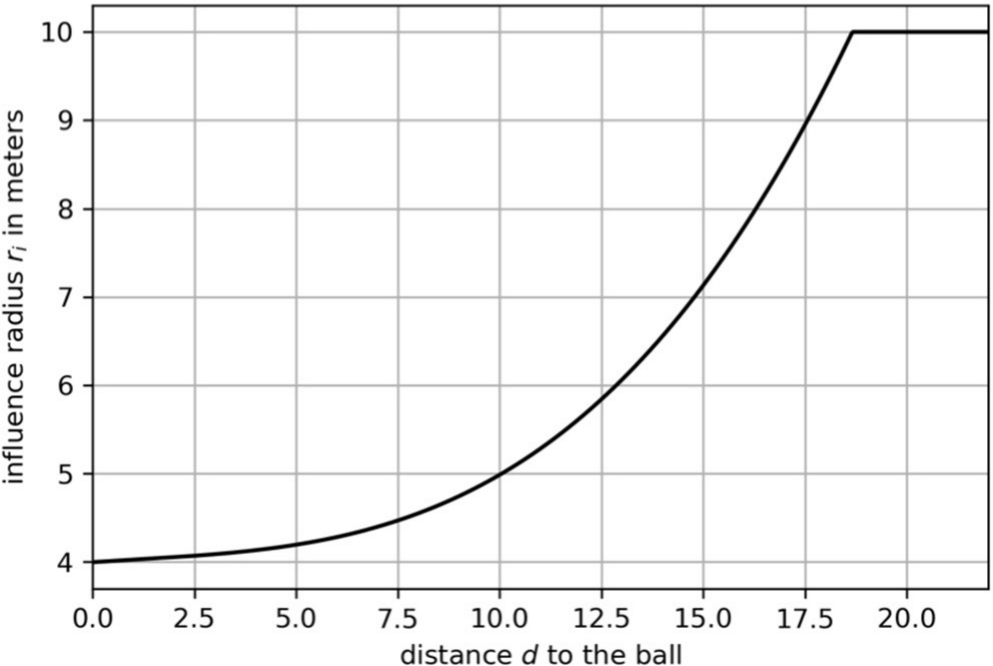
A function that maps the distance *d* to the influence radius *r*^*i*^.

The mean $\mu_{t}^{i}$ of $f_{t}^{i}$ is given by the position of the player and his velocity vector ${\text{v}}_{t}^{i}$ using

$$\begin{array}{l} \mu_{t}^{i}={\text{p}}_{t}^{i}+\frac{1}{2}\cdot {\text{v}}_{t}^{i} \end{array}$$

where ${\text{v}}_{t}^{i}$ is defined by

$$\begin{array}{l} {\text{v}}_{t}^{i}={\text{p}}_{t}^{i}-{\text{p}}_{t_{\delta }}^{i}=\left(x_{t}-x_{t_{\delta }},y_{t}-y_{t_{\delta }}\right) \end{array}$$

with *t*_δ_ = *t* − δ for an arbitrary time difference δ > 0. The covariance matrix $\Sigma_{t}^{i}\in {\mathbb{R}}^{2\times 2}$ is a function of the velocity and distance of a player to ball, as shown in Figure 1. Its computation resembles an eigendecomposition and is given by

$$\begin{array}{l} \Sigma_{t}^{i}={\text{R}}_{t}^{i}{\text{V}}_{t}^{i}{\text{V}}_{t}^{i}{\left({\text{R}}_{t}^{i}\right)}^{-1} \end{array}$$

where **R** is the rotation matrix that twists the bivariate normal counterclockwise according to the direction of the velocity vector

$$\begin{array}{l} \text{R}=\left[\begin{array}{cc} \cos\theta & -\sin\theta \\ \sin\theta & \cos\theta \end{array}\right] \end{array}$$

with $\theta =atan2\left(y_{t}^{i}-y_{t_{\delta }}^{i},x_{t}^{i}-x_{t_{\delta }}^{i}\right)$. Finally, the scaling matrix ${\text{V}}_{t}^{i}$ determines the area of the distribution by

$$\begin{array}{l} {\text{V}}_{t}^{i}=\left[\begin{array}{cc} \frac{r_{t}^{i}+\left(r_{t}^{i}{\left(\frac{{\text{v}}_{t}^{i}}{v_{max}}\right)}^{2}\right)}{2} & 0\\ 0 & \frac{r_{t}^{i}-\left(r_{t}^{i}{\left(\frac{{\text{v}}_{t}^{i}}{v_{max}}\right)}^{2}\right)}{2} \end{array}\right] \end{array}$$

where radius *r*^*i*^ depends on the Euclidean distance between a player ${\text{p}}_{t}^{i}$ and the ball ${\text{p}}_{t}^{b}$. By referring to expert knowledge, the authors restrict *r*_*i*_ to be in a range of [4, 10] meters. This function^1^ is shown in Figure 2. The quantity *v*_*max*_ is the maximum speed of all the players. We refer to Fernandez and Bornn (2018) for more details on *r* and *v*_*max*_. Figure 1 shows two exemplary situations to illustrate how velocity and distance to the ball affect the shape of the Gaussian. Note that this approach ignores movement capabilities of an individual, e.g., agility and acceleration.

### 3.3. Data-Driven Movement Models

Influence on the pitch can also be determined directly by possible movements of players in the near future. One could argue that a player can only influence the area she can actually reach in a given time window. While many movement models have been proposed by approximating equations from physics, Brefeld et al. (2019) present a data-driven movement model by computing frequency statistics from historic games. Their approach leads to individual player movement models that capture characteristic traits of the respective player.

The approach grounds on triplets $\left({\text{p}}_{t_{\delta }}^{i},{\text{p}}_{t}^{i},{\text{p}}_{t_{\Delta }}^{i}\right)$ generated by the *i*-th player, with *t*_δ_ = *t* − δ for a time horizon *t*_Δ_ = *t* + Δ such that δ, Δ > 0 and *t*_δ_ < *t* < *t*_Δ_ holds. Each triplet is a subset that represents the trajectory of a player with past, current, and final position. Hence, ${\text{p}}_{t_{\delta }}^{i}$ and ${\text{p}}_{t}^{i}$ can be used to estimate the velocity vector ${\text{v}}_{t}^{i}$ including the direction a player is heading to at time *t*. Given this initial velocity, ${\text{p}}_{t_{\Delta }}^{i}$ represents a point that a player is able to reach in Δ time steps. To this end, all triplets of the same player are mapped (and rotated) into a new coordinate system such that the first part realizes a movement along the *x*-axis and the final endpoints of the triplets indicate points that are reached by the player in time Δ with initial velocity *v* given by the Euclidean norm of the velocity vector ||**v**||_2_ [see Brefeld et al. (2020) for details on how to estimate *v* from tracking data]. To be concrete, ${{\text{p}}^{\prime }}_{t_{\Delta }}$ is given by

(1) $$\begin{array}{l} \left(x_{t_{\Delta }}^{\prime },y_{t_{\Delta }}^{\prime }\right)=\left(d\cdot \cos\theta ,d\cdot \sin\theta \right) \end{array}$$

where the rotation angle θ is computed as above,

(2) $$\begin{array}{l} \theta =∡\left(\vec{{\text{p}}_{t_{\delta }}{\text{p}}_{t}},\vec{{\text{p}}_{t}{\text{p}}_{t_{\Delta }}}\right)\\ \;=atan2\left(y_{t}-y_{t_{\delta }},x_{t}-x_{t_{\delta }}\right)-atan2\left(y_{t_{\Delta }}-y_{t},x_{t_{\Delta }}-x_{t}\right), \end{array}$$

and distance *d* is defined by

(3) $$\begin{array}{l} d={\left\|\vec{{\text{p}}_{t}{\text{p}}_{t_{\Delta }}}\right\|}_{2}. \end{array}$$

To obtain an individual movement model for player *i*, all available triplets $\left({\text{p}}_{t_{\delta }}^{i},{\text{p}}_{t}^{i},{\text{p}}_{t_{\Delta }}^{i}\right)$ are extracted from historic games and transformed according to the above procedure. The resulting endpoints are collected together with the initial velocities in a set. This can be carried out for each Δ in a finite set of time horizons T such that the result is $S_{\Delta \in T}^{i}=\left\{\left({\text{p}}_{t_{\Delta }}^{i},v_{t}\right)\right\}$. The time window δ to obtain the initial velocity vector remains fixed for all combinations. For practical reasons, similar velocities are often aggregated into bins of similar ranges. Since all passes are completed within 5 s, we use the time horizons T={0.2,0.4,…,5}. The initial velocity is estimated in the preceeding δ = 0.2 s. Following Brefeld et al. (2019), we group velocity ranges into standing ([0, 1) km/h), walking ([1, 7)), jogging ([7, 14)), running ([14, 20)), and sprinting (≥ 20). Every triplet in the same bin is then summarized by a non-parametric kernel density estimation (KDE)^2^ with Gaussian kernel as it seems to be a good fit for the resulting endpoint distributions. The bandwidths of the kernels are optimized using Bayesian optimization (Brochu et al., 2010; Snoek et al., 2012; Srinivas et al., 2012). We denote the resulting probability density by ${\mathbb{P}}_{\Delta }^{i}\left(\text{p}|{\text{p}}_{t_{\delta }}^{i},{\text{p}}_{t}^{i},v_{t}^{i}\right)$. The measure ${\mathbb{P}}_{\Delta }^{i}$ computes the probability density that player *i* can reach position **p** in time Δ from position ${\text{p}}_{t_{\delta }}^{i}$ with initial velocity $v_{t}^{i}$.

**Figure 3:**
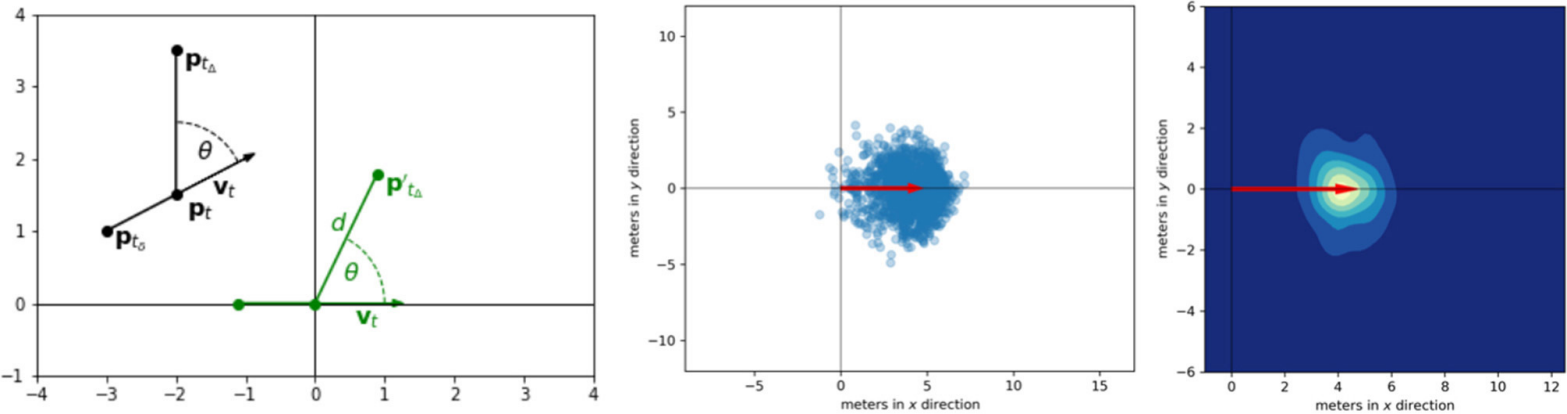
Data-driven movement models. **(Left)** The initial position *p*_*t*_ is mapped to the origin such that the initial direction of movement *v*_*t*_ follows the x-axis. Position *p*_*t*_Δ__ marks the end of the trajectory. Position *p*_*t*_δ__ is required to estimate initial velocity. **(Center)** Resulting point cloud. **(Right)** Smoothed movement model using density estimation.

Figure 3 shows an example: Trajectories of players are projected into a new coordinate system such that every trajectory starts in the origin with an initial movement along the *x*-axis. The endpoints of the trajectories are then stored for the actual initial velocity and time window. Depending on the application, the point distribution can be either used directly or approximated by its convex hull. We refer to Brefeld et al. (2019) for details on the computation of data-driven movement models.

## 4. Quantifying Space

### 4.1. Influence of the Player

Fernandez and Bornn (2018) introduce PC to measure the dominance of players and teams in certain areas on the pitch. In that sense, PC is similar to *dominant regions* (Taki et al., 1996) or *zones of control* (Brefeld et al., 2019). We aim to study data-driven movement instead of Gaussian approximations together with PC.

For the data-driven approach, we need to map the distance between player and ball to a time horizon Δ. Since our analyses will focus on passing events, the amount of time a player can move around is upper bounded by the time it would take to pass the ball to her. This can directly be translated into the time horizon that is necessary to select the best-suited player probability density of the player ${\mathbb{P}}_{\Delta }^{i}$ because of the binning into discrete time intervals Δ∈T. This function can also be learned from historic data using pass data as an approximation. The idea is to learn a predictor of the time a player usually has to reach the ball given the initial distance between him and the passer at the time the pass was initiated *t*_*p*_. For example, for short distances the receiving player has less time to react and therefore less ground she can cover to get herself in an open-spot position to receive a pass. The distance is then defined as the Euclidean norm of the vector between passer **p**^*b*^ and receiver **p**^*r*^ at time *t*_*p*_:

$$d=∥\vec{{\text{p}}_{t_{p}}^{b}{\text{p}}_{t_{p}}^{r}}{∥}_{2}.$$

**Figure 4:**
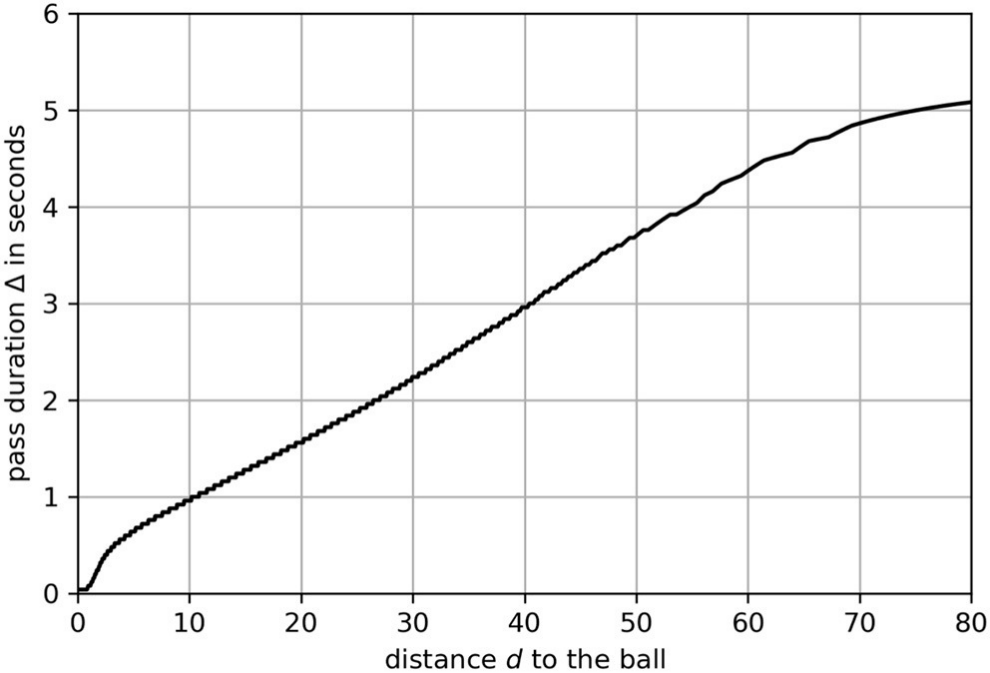
The function that maps the distance *d* to time window Δ.

The time window Δ is derived by the duration of pass i.e., the traveling time of ball from passer to receiver

$$\Delta =t_{r}-t_{p}$$

with *t*_*r*_ being the point in time, the receiving player actually receives the ball^3^. The mapping function can be phrased as a regression task with Δ as response and *d* as an explanatory variable. We use 25,663 passes to calculate the distance *d* and pass duration Δ. In short, 80% of the passes are used as the training set. Hyper-parameters are optimized using cross-validation. All models are finally validated against the remaining 20% of the passes, and the best model is chosen by selecting the one with the minimal mean squared error on the validation set. The resulting linear regression^4^ provides a power feature transformation (Yeo and Johnson, 2000) to better fit the underlying assumptions for linear regression models (e.g., homoscedasticity in errors). The learned relationship between distance and time is shown in Figure 4.

Finally, influence likelihoods of the players are normalized such that the degree of control of a player's for each point on the field lies in the interval [0, 1] by dividing the likelihood of each point **p**^*j*^ with the likelihood at the underlying mode of distribution (main). This will further be referred to as the *player influence area* (PI). For data-driven movement models, the main mode is computed with mean shift (Comaniciu and Meer, 2002), and the PI is given by

(4) $$\begin{array}{l} PI_{t}^{i}\left(\text{p}\right)=\frac{{\mathbb{P}}_{\Delta }^{i}\left(\text{p}|{\text{p}}_{t_{\delta }}^{i},{\text{p}}_{t}^{i},v_{t}^{i}\right)}{{\mathbb{P}}_{\Delta }^{i}\left(mode|{\text{p}}_{t_{\delta }}^{i},{\text{p}}_{t}^{i},v_{t}^{i}\right)} \end{array}$$

As a result, the influence value of the player at the main mode (the highest peak) of each movement distribution has the value $PI_{t}^{i}=1$^5^.

### 4.2. Pitch Control

*Pitch control* (PC) for a team is defined as the sum over all influence areas of players. Hence, with all players belonging to team *a* collected in set A and their opponents in set B, the summed team influences can be subtracted to obtain the pitch control at point **p** at time *t*,

(5) $$\begin{array}{l} PC_{t}\left(\text{p}\right)=\sigma (\underset{a\in A}{\sum }PI_{t}^{a}\left(\text{p}\right)-\underset{b\in B}{\sum }PI_{t}^{b}\left(\text{p}\right)), \end{array}$$

where σ maps *PC* into an appropriate interval. In the remainder, we make use of tanh : ℝ ↦ [−1, 1], i.e., the value *PC*_*t*_(**p**) = −1 indicates that the defensive team has full control at point **p** and time *t* whereas *PC*_*t*_(**p**) = 1 means that the offensive team controls that area.

**Figure 5:**
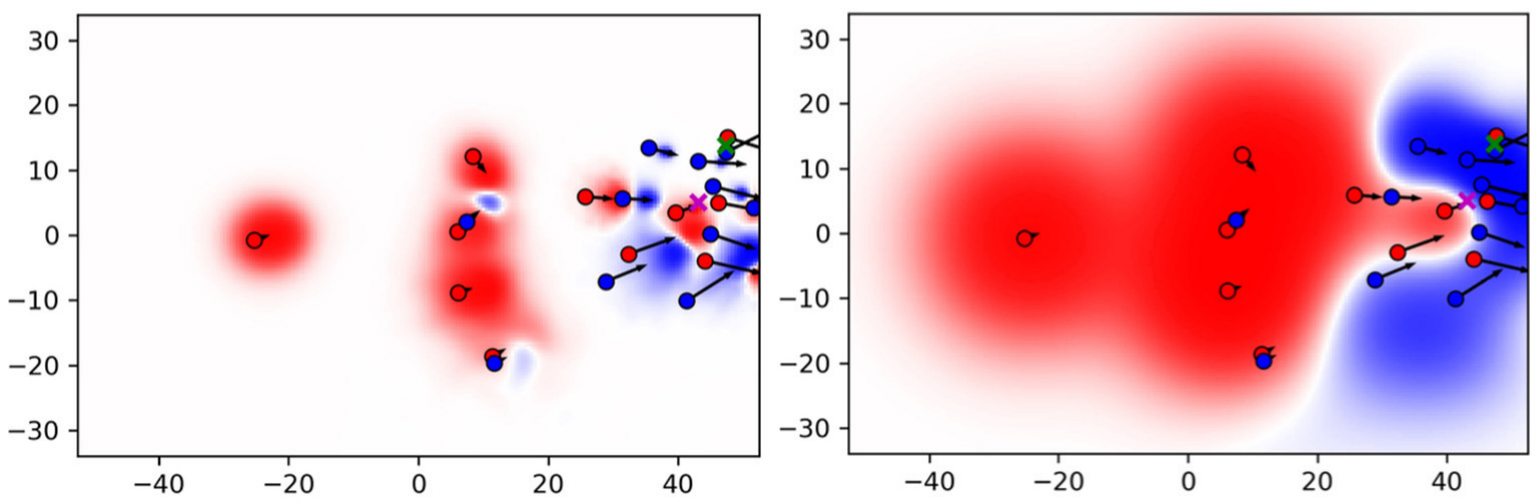
Pitch control (PC) for the proposed approach **(Left)** and baseline **(Right)**.

Figure 5 compares the resulting PC with the original formulation in Fernandez and Bornn (2018) (baseline) on an exemplary situation. The red team plays from left to right. The ball is currently at the green cross and being passed to the purple cross where the red striker scores with a volley shot. The figure reveals the main differences between both the approaches. The influence areas of the baseline are much larger and cover a great deal of the pitch. By contrast, influence areas computed with the data-driven movement model are much smaller, especially when a player is close to the ball. Note that the location of the purple cross has a PC value of –0.21 for the baseline, while the proposed approach clearly reflects the known outcome of this scoring possession by a PC value of 0.57. Since the red player is already in possession of the ball and moreover able to pass it on to the striker, the data-driven model delivers a more realistic interpretation of control on the pitch.

**Figure 6:**
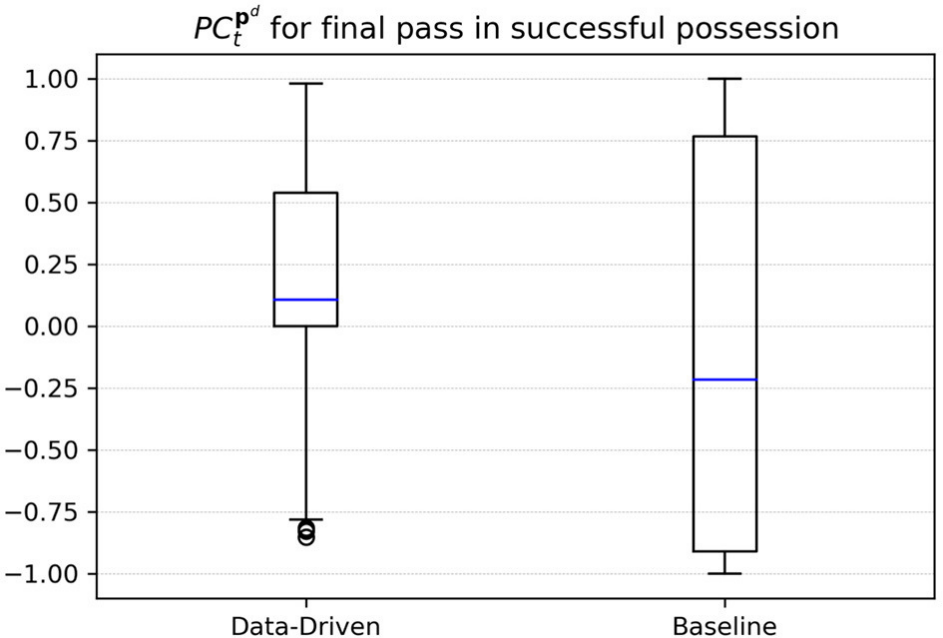
PC for final passes before a shot was made.

To confirm this impression, we aim to conduct an experiment on all 289 successful ball possession phases in the data. Throughout this analysis, we define a possession to be successful if it ends with a shot at the goal. We focus on sequences with at least three successful passes because the vast majority of possessions with fewer passes are rather chaotic and, e.g., consist of a series of headers after a goal-kick. Analog to the example above, we collected the pitch control $PC_{t}^{{\text{p}}^{d}}$ for the attacking team at the pass destination **p**^*d*^ and at the time *t* the final pass was made before the attacker shots at the goal. Figure 6 compares the results of both models. For our data-driven approach, in nearly 75% of the cases the attacking team has a positive PC before the pass receiver is able to take a shot. This follows the intuition that the attacking team must have created some space to realize the shot at the target. Using the baseline model, however, the observed PC values do not allow for an informed guess on the known outcome of these situations.

### 4.3. Pitch Value

While PC provides interesting insights, many of the colored regions in Figure 5 are irrelevant for the shown situation (e.g., space controlled by the red goal-keeper). Again, we borrow concepts from Fernandez and Bornn (2018) to compute the value of a position. The underlying idea is that defensive players intuitively cover highly valuable space. Obviously, defensive players do not position themselves perfectly in every situation, e.g., to prevent through-passes. But we argue that such individual mistakes are exceptions and that defenders usually cover the important areas on the pitch in similar situations, hence, with a sufficiently high number of training situations that a model should be able to generalize well and predict the high valued space.

We thus aim to learn influence areas for a defending team from historic data given the ball position at that time ${\text{p}}_{t}^{b}$. This will be referred to as *defensive influence* (DI). The observed DI on point **p**^*j*^ is the sum of influences of all players in the defensive team A at time *t*,

(6) $$\begin{array}{l} DI_{t}^{{\text{p}}^{j}}\left({\text{p}}_{t}^{b}\right)=\min\left\{\underset{a\in A}{\sum }PI_{t}^{a}\left({\text{p}}^{j}\right),1\right\}. \end{array}$$

Analogous to PC, we define the maximum amount of DI to be one. Using this definition, the *pitch value* is defined as

(7) $$\begin{array}{l} PV_{t}^{{\text{p}}^{j}}\left({\text{p}}^{b}\right)=\left(1-\frac{{\left\|\vec{{\text{p}}^{j}{\text{p}}^{g}}\right\|}_{2}}{{\left\|\vec{{\text{p}}^{c}{\text{p}}^{g}}\right\|}_{2}}\right)\cdot DI_{t}^{{\text{p}}^{j}}\left({\text{p}}_{t}^{b}\right). \end{array}$$

Here, **p**^*c*^ denotes the point at the opposite corner such that the denominator marks the longest possible distance to the goal. Hence, pitch value equals the defensive influence scaled by the distance to the goal that is in the range [0, 1] following the idea that points on the pitch are generally more valuable the closer they are to the goal of the opponent.

Though $DI_{t}^{{\text{p}}^{j}}$ can be extracted from historic games, we need a function $fn_{\theta }\left({\text{p}}_{t}^{b},{\text{p}}^{j}\right)$ that approximates $DI_{t}^{{\text{p}}^{j}}$ well and that can be applied to new and unseen situations for generality. Following Fernandez and Bornn (2018), we propose to learn *fn*_θ_ with a feed-forward neural network (FNN) by minimizing the mean squared error,

$$\begin{array}{l} \underset{\theta }{\min}\underset{t,{\text{p}}^{j}}{\sum }(DI_{t}^{{\text{p}}^{j}}\left({\text{p}}_{t}^{b}\right)-fn_{\theta }\left({\text{p}}_{t}^{b},{\text{p}}^{j}\right){)}^{2}. \end{array}$$

The training data contain game situations from 54 Bundesliga games (about 34 million observations) where goalkeepers are ignored. To render this training task computationally feasible, we choose points as features that lie on an equally spaced 21 × 16 grid G such that ${\text{p}}^{j}\in G$. This results in a (|T|×|G|)×4 feature matrix **X** where each row contains the (*x, z*) coordinates of one ${\text{p}}^{j}\in G$ and the ball position ${\text{p}}_{t}^{b}$ at time *t* for all available timestamps T in the data set. Dropout (Srivastava et al., 2014) is applied to all hidden layers to prevent over-fitting, and all hyper-parameters (# layers, # units per layer, dropout rate, and learning rate batch size) of the network are optimized with Bayesian optimization. In our experiments, the network with the best performance had two hidden layers and 64 units in each layer. The optimization was carried out using the Adam optimization algorithm (Kingma and Ba, 2015).

**Figure 7:**
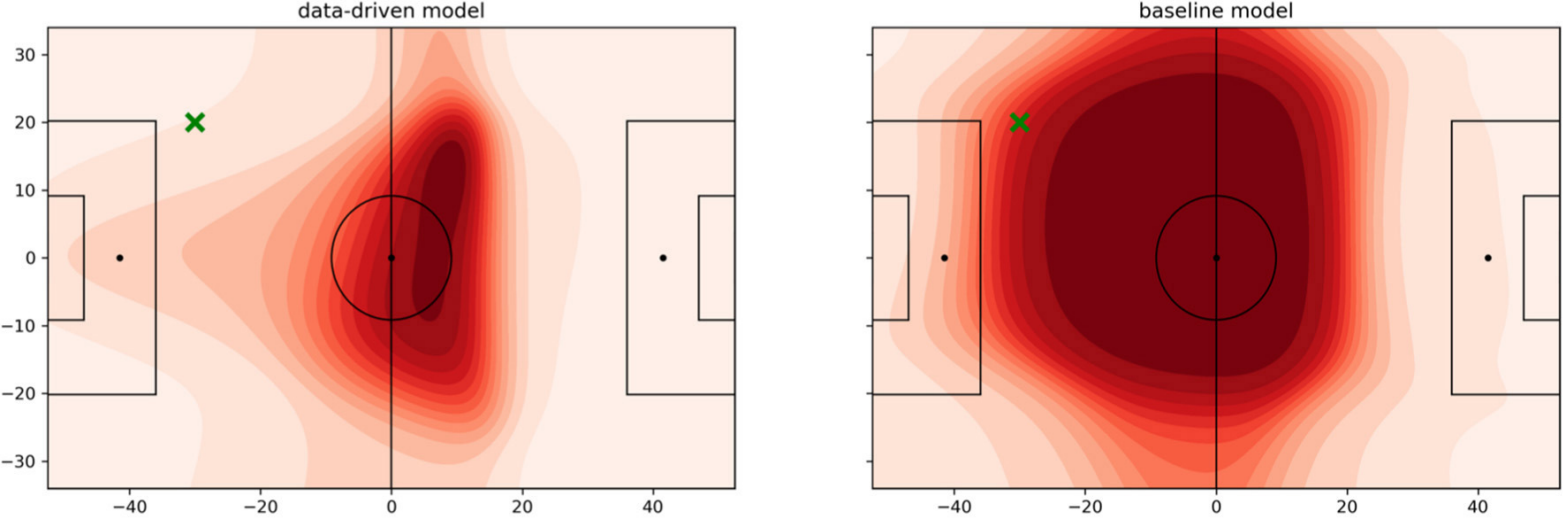
Pitch value using the learned function *fn*_θ_. The ball is located at the green cross, and the attacking team plays from left to right. **(Left)** data-driven. **(Right)** baseline.

Figure 7 shows an example of the data-driven approach and the baseline (Fernandez and Bornn, 2018). The ball is on the left wing just outside the box visualized by the green cross. The attacking team plays from left to right. In the data-driven model, the last defending line forms up right behind the center-line with the intention to use the offside rule to limit the space in which the attacking team can operate. The right defender covers space slightly deeper than his peers on the left side. This is a useful tactic to prevent straight and long passes in the back of the last defending line. It also discloses the habit of defending teams to prevent crosses from one side to the other. Strikers and offensive midfielders position themselves in a way that their opponent is forced to play the long passes mostly along the sideline. The influence area reaches far out to the left penalty box to isolate the ball-possessing player on his side. Such insights are hidden in the results of the baseline that considers about half of the pitch important.

### 4.4. Space Quality

As shown in the previous sections, pitch control measures the amount of dominance that a player or team has on a certain location. Pitch value, by contrast, relates to the value that a location has at that very moment. *Space quality* (SQ) for the *j*th location at time *t* is now simply defined as the product of pitch control and pitch value (Fernandez and Bornn, 2018),

(8) $$\begin{array}{l} SQ_{t}^{{\text{p}}^{j}}=PC_{t}^{{\text{p}}^{j}}\cdot PV_{t}^{{\text{p}}^{j}}\left({\text{p}}_{t}^{b}\right). \end{array}$$

**Figure 8:**
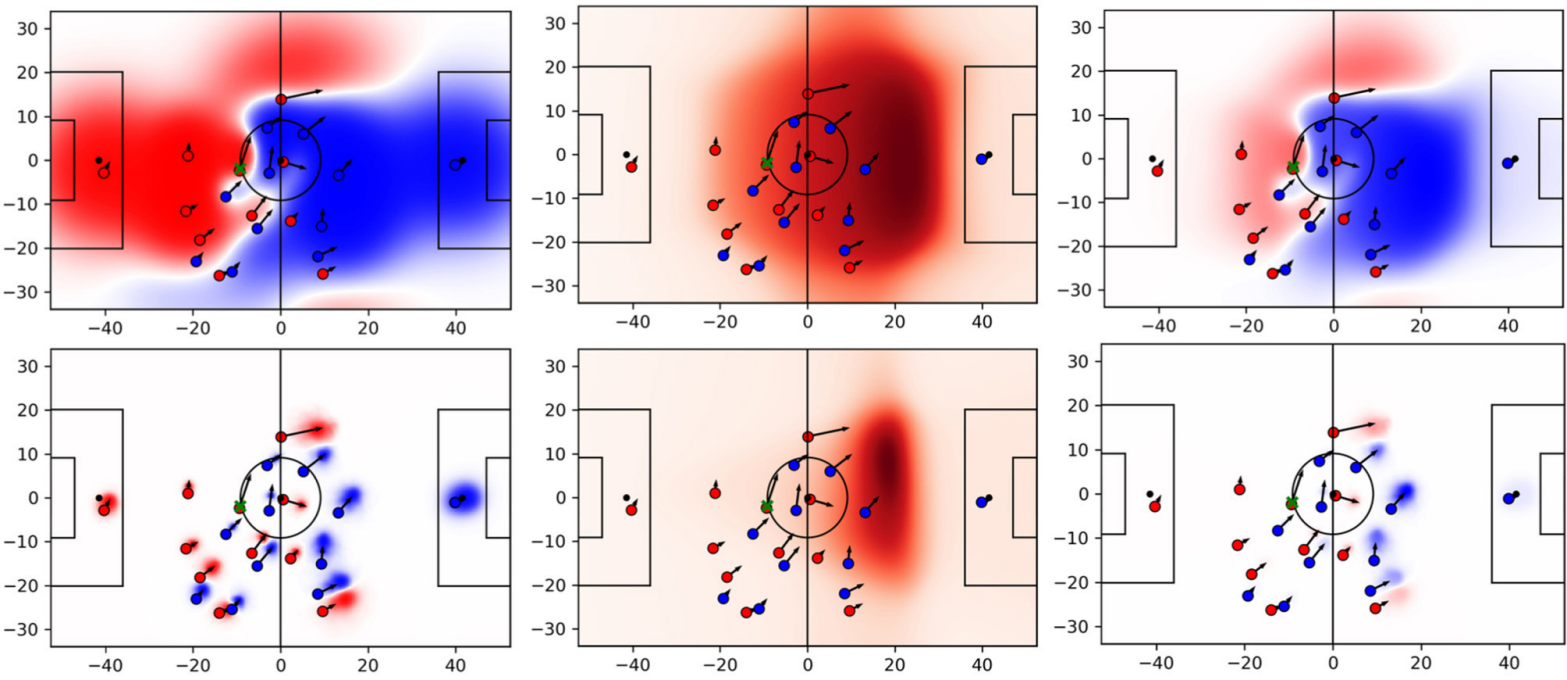
PC **(left column)**, pitch value **(center column)**, and space quality **(right column)** for baseline **(top row)** and proposed approach **(bottom row)**.

Figure 8 shows all three parts of the equation for the same situation. The red team stages an attack that, later on, ends up in a shot at goal. The player with the ball (green cross) plays a deep forward pass to the red player on the left wing. The pass receiver generates pitch control in a highly valuable area that results in space of high quality. Note the differences of the two models around the left winger. The baseline credits much space in her back to her team due to an excessively large influence of the player. However, given the velocity vector of a player in this situation the player can hardly control the areas behind her, especially given the rather short distance to the passer. Moreover, the baseline estimates the area directly in front of her as a neutral zone (white). With the data-driven model, however, that particular area turns dark red as one would expect in this situation. Also note that the defensive team is pretty disorganized; they are not doing well in covering the important space because their defensive line and especially their right back moved up too far that allows the aforementioned left attacker to run into the exposed region.

## 5. Space Generation

While the previous section suggests that identifying the impact of the player with individual movement models actually makes sense, we now turn toward establishing an empirical basis for this insight. Since the devised quantities are difficult to evaluate quantitatively, we resort to proxies and study space generation and measurable outcomes of ball possession phases.

To connect to the previous section, we first test the hypothesis that passes into areas of high space quality are more likely to result in a positive outcome than passes into zones with small space quality. We follow a simple setup: For each pass in the event log, the resulting space quality is computed at equidistant points ${\text{p}}^{j}\in F$ lying on a 50 cm-spaced grid over the pitch^6^. We use only two predictors: (i) the average space quality at the location of the passer **p**^*o*^ and (ii) the average space quality at the position of the pass destination **p**^*d*^ for every possession. To take the distance between a position (grid cell) and the pass origin and destination, respectively, as well as some smaller inaccuracies in the pass event data into account, we weigh space quality with exponential decaying factors λ^*o*^ and λ^*d*^, so that positions far away from the pass origin and destination, respectively, do not impact the results. The magnitude of the exponential decay is controlled by parameters that are found by model selection. So, the features for the *k*th possession with *n*_*p*_ pass timestamps $T_{k}$ are defined as:

(9) $$\begin{array}{l} x_{o}^{k}=\frac{1}{n_{p}}\underset{t\in T_{k}}{\sum }\underset{{\text{p}}^{j}\in F}{\sum }SQ_{t}^{{\text{p}}^{j}}\cdot \exp\left(-\lambda^{o}\cdot {\left\|\vec{{\text{p}}^{j}{\text{p}}^{o}}\right\|}_{2}\right) \end{array}$$

(10) $$\begin{array}{l} x_{d}^{k}=\frac{1}{n_{p}}\underset{t\in T_{k}}{\sum }\underset{{\text{p}}^{j}\in F}{\sum }SQ_{t}^{{\text{p}}^{j}}\cdot \exp\left(-\lambda^{d}\cdot {\left\|\vec{{\text{p}}^{j}{\text{p}}^{d}}\right\|}_{2}\right) \end{array}$$

We use 5,277 ball possession phases in 54 Bundesliga matches containing 31,824 passes where episodes with fewer than three passes are discarded. In sum, 5.5% of the remaining data constitute successful ball possession phases that end with a shot at goal. These form the positive class. We use a linear support vector machine (SVM) to learn a model that predicts whether an attack is successful or not, based on the two input features.

**Figure 9:**
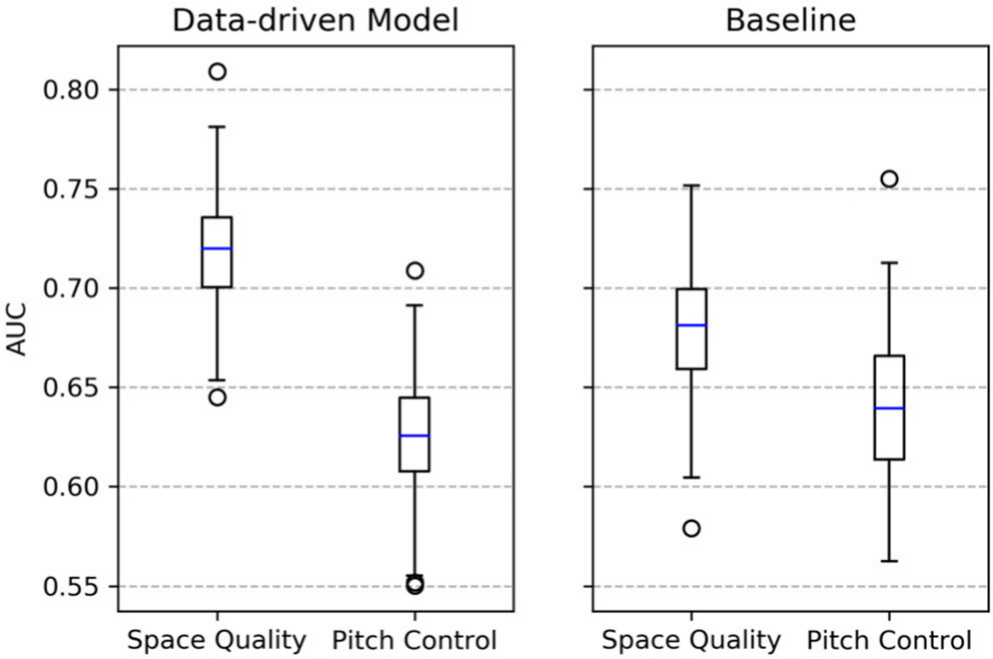
The resulting area under the curve (AUC) values.

For each experiment, we randomly choose 80% of the data for training and 20% for model evaluation using area under the curve (AUC). For every combination of parameter and model, we repeat the experiment 1,000 times. To analyze the effect of adding pitch value to the space quality equation, we repeat the same setup but replace space quality with pitch control in Equations (9) and (10). The results are shown in Figure 9. Using only pitch control does not lead to significant differences between data-driven and baseline approaches. However, adding pitch value and thus focusing on space quality lead to a much better predictive accuracy for the data-driven approach.

For the data-driven approach, the classifiers perform even better: A very fine-grained focus on the pass destination increases the ability to predict the outcome of the ball possession. Translated to the situation in Figure 5, an area in a radius of 1.5 m around the shot position is considered as sufficient for the classifier. This area is largely controlled by the red team. The detailed focus on a small area around the pass destination is possible because the data-driven model is able to approximate pitch control more accurately than the baseline does. This is reflected by pitch control values at the shot location ($PC_{t}^{{\text{p}}^{d}}=0.57$ for the data-driven model vs. $PC_{t}^{{\text{p}}^{d}}=-0.21$ for the baseline model) and in Figure 6. In fact, for the baseline the classification results show a very different behavior: the smaller the considered space, the worse the performance. Overall, the classifiers based on the data-driven model significantly outperform the ones that ground on features from the baseline model. Often, large average space quality values in ball possession phases are caused by only a few high-quality passes.

Unsurprisingly, these experiments show that it is beneficial for a soccer team to create valuable space during a possession through passing in order to get in promising situations to score a goal. Our analysis confirms that this can actually be measured with the proposed approach. Our approach turns out accurate and allows to derive meaningful metrics for individual players.

### 5.1. Measuring the Generation of Space

We now leverage space quality to off-ball movements and space generation. A simple way to measure the off-ball movement is to compute space quality $SQ_{t}^{i,\text{p}}$ for player *i* at time *t* and location **p** and subtract the space quality of all other players j∈P\{i} at that point and time,

(11) $$\begin{array}{l} SG_{t}^{i}=\underset{\text{p}\in F}{\sum }\underset{j\in P\backslash \left\{i\right\}}{\sum }max\left\{\left(SQ_{t}^{i,\text{p}}-SQ_{t}^{j,\text{p}}\right),0\right\}. \end{array}$$

Hence, the resulting *space generation* is the sum of individual space quality over an equally spaced grid F, i.e., the amount of control that this player actually has on certain areas on the pitch weighted by the pitch value. Note that this measurement approach differs from the *space generation gain* concept in (Fernandez and Bornn, 2018), which quantifies the space that an attacker frees up by dragging the opponents into his direction.

To compute the rating of an individual player for space generation, $SG_{t}^{i}$ is evaluated for all timestamps at which an offensive player controls the ball and attempts to make a pass. The following analysis is based on data from six teams playing against each other leading to a subset of 30 games with a total of 16,631 passes. Space generation is again computed on a 50 cm-equidistant grid on the pitch. In our analysis, we only consider the 98 players who were involved in at least 30 passes (either as passer or pass receiver) during these games for a robust comparison.

In the remainder, *SG*_*rec*_ denotes the amount of average space created by a pass receiver and *SG*_*pas*_ credits this amount to the passing player. *SG*_*rec*_ thus corresponds to a player creating space for herself by positioning in areas where she can get the ball. Similarly, *SG*_*pas*_ describes the ability of a passer to identify valuable spaces and to pass the ball into valuable areas that were generated by her teammates. *SG*_*total*_ simply defines the sum of both measurements.

We focus on possible relationships between our space generation metrics and existing player metrics and valuations. Prominent concepts are the *expected goal* (xG) and *expected assists* (*xA*) metrics that measure the probability that whether a shot will result in a goal and credit this likelihood either to the shooter (*xG*) or the pass giver (*xA*), respectively. Although implementations differ in details, the basic idea is to compare shots with similar characteristics (e.g., shot position and body part the attacker made the shot with) and calculate how many of these shots actually resulted in a goal (Lucey et al., 2015; Le et al., 2017; Rathke, 2017). Besides its popularity, we choose these measures because, compared to the actual number of goals, it leaves aside factors such as luck and rather aims at the ability of the players to bring herself into situations to score^7^. From that point of view, *xG* and *SG*_*rec*_ pursue similar goals as the latter values the ability of a player to bring herself into a position to receive passes in high-quality areas that ultimately (for the final pass in a possession) results in a position to shoot at the goal.

**Figure 10:**
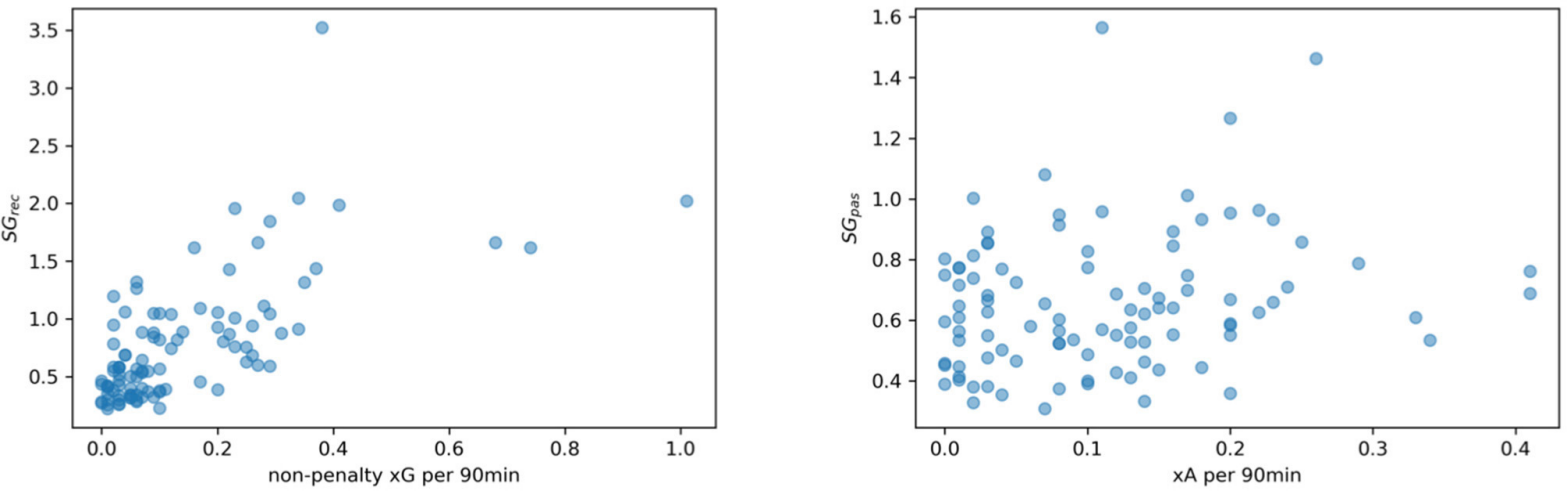
Correlation between *SG*_*rec*_ and non-penalty *xG* per 90 min **(Left)** and between *SG*_*pas*_ and *xA* per 90 min **(Right)**.

Figure 10 (left) clearly shows a significant positive correlation between both metrics [Pearson's *r* = 0.66 with *p*-value = 8.85e − 14 and CI = (0.54, 0.76)]. For a more meaningful comparison, the *xG* value is standardized per 90 min and penalty kicks are excluded^8^. Note that the result is almost unaffected by the three players with *xG* > 0.6 and the one with *SG*_*rec*_ > 3 [*r* = 0.66, *p*-value = 7.49e − 13, CI = (0.52, 0.76)]. These four players are strikers with very high *SG*_*rec*_ values, so all of them are able to create high valued space. In addition, the three players with a superior *xG* > 0.6 are exceptionally good in converting shots into goals. For the player with *SG*_*rec*_ = 3.52, the story is quite different. Despite the outstanding ability to create high valued space, this player is often unable to convert these situations.

Figure 10 (right) shows the results for *SG*_*pas*_ and *xA*. Although their relation is not as strong as in the previous comparison, their correlation is still positive and significant [Pearson's *r* = 0.21, p-value = 0.03, CI = (0.02, 0.4)]. This confirms our initial intuition that both concepts describe similar aspects of the game. Space generation metrics are not limited to shot or scoring events but allow also for useful insights on preceding actions in ball possessions and game analyses, as we will see in section 5.2. This becomes clear, in particular, for the *SG*_*pas*_ and *xA* comparison. On one hand, *xA* only accounts for the direct pass before a shot even though the more important pass might have been the one to initiate the attack. As mentioned above, the receiver metric *SG*_*rec*_ does not give any insights on how well the controlled space is used, i.e., the decision-making or the cognitive and physical skills after receiving the ball. On the other hand, *xG* neglects the amount of defensive pressure; hence, shots can have a high value even though the attacker is well covered by the defenders.

### 5.2. Game Analyses

**Figure 11:**
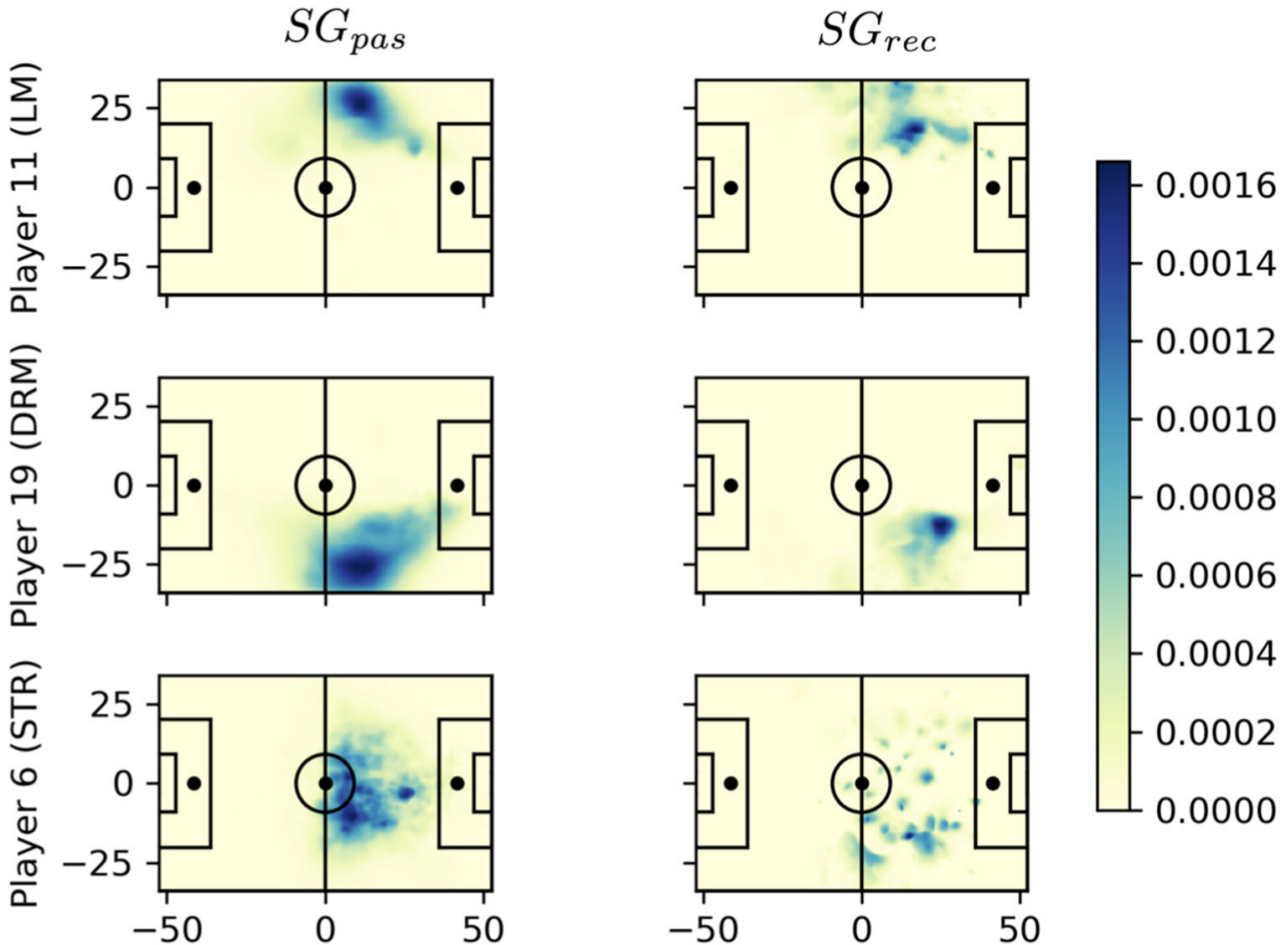
Comparison of average space quality of the player at the time of all passes **(left column)** and at the time each player is the actual pass recipient **(right column)**. Playing direction is from left to right. The *SG* values in the color bar relate to the average space quality a player generates at a certain field position during all considered passing events.

In this section, we aim to sketch an application of our contribution to the data-driven analysis of games. The central idea is to identify dangerous passes and the corresponding pass givers and receivers and to aggregate this information over historic data. Clearly, there are additional factors for players to decide where to pass the ball, such as technical skills and crowded passing lanes. Hence, as a pass receiver, it is important to not only generate space but also ensure a positioning that actually allows to receive the ball. Figure 11 compares the average space quality created by three players for all their passes (left) and received balls (right). The midfielders in the first two rows show clear areas of high quality on the left and right wings, respectively. In particular, player 19 has the highest average space quality as a pass receiver. When receiving the ball, he creates space much closer to the area in front of the penalty box than player 11 although both usually control space next to the center-line. As a pass receiver, he creates space everywhere in the opponent's half and is particularly difficult to defend.

**Figure 12:**
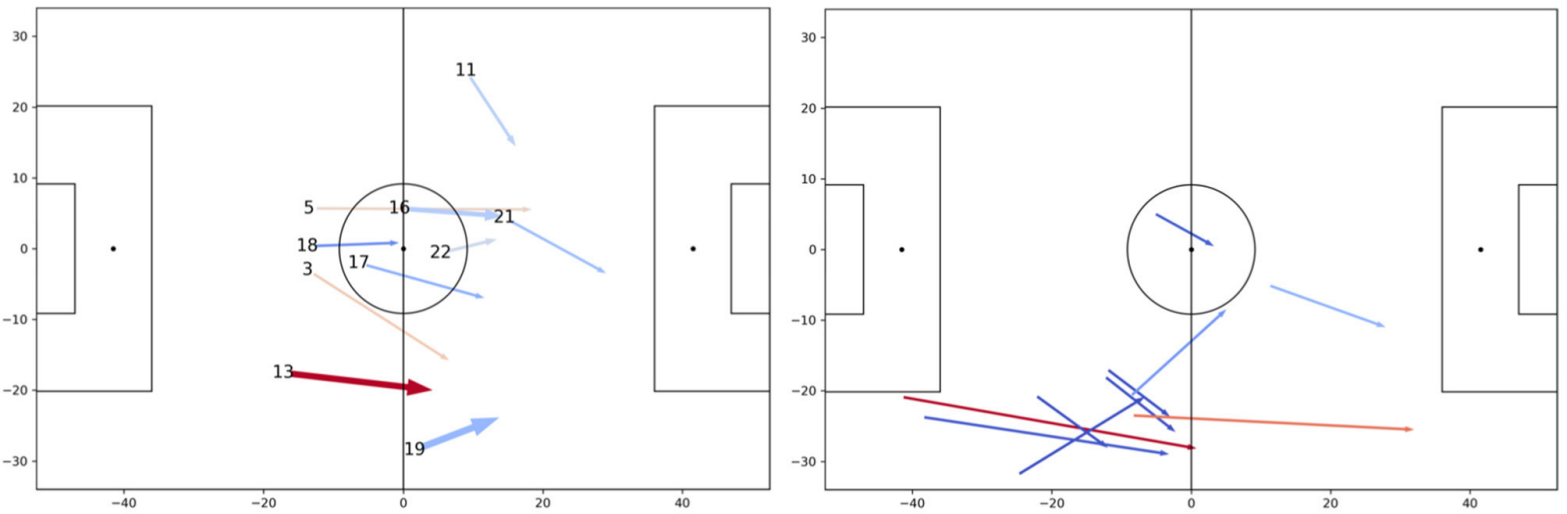
**(Left)** Average passes to player 6. **(Right)** Passes from 13 to 6.

For a more detailed view, we choose this player number 6 (cmp. Figure 11 bottom row) because of his widely distributed space generation pattern and his high *SG*_*pas*_ = 0.71 and *SG*_*rec*_ = 1.315 scores. We focus on his receiving qualities and aim to understand where these passes come from and, optimally, from which locations and/or player. Figure 12 (left) shows aggregates of all passes to player 6 in that game, summarized by his teammates. Displayed are also the number of passes by arrow width and the average *SG*_*rec*_ values by color. The color legend ranges from light blue (low *SG*_*rec*_) to dark red (high *SG*_*rec*_). The figure clearly singles out player 13 as the teammate who creates space of high value by his passes to player 6. Although the overall *SG*_*pas*_ metric for player 13 is only average, his passes to player 6 are exceptional.

Figure 12 (right) zooms in on this particular connection between the two player. All ten passes from player 13 to 6 are shown by arrows where the color is drawn from the legend before, especially two long passes along the sideline result in very high *SG* values. Also, the third long ball generates space above average. Based on this brief analysis, long passes from 13 to 6 must be prevented by the opposing team to decrease the dangerousness of striker 6. Particularly when both players are acting on the right side of the pitch, the other team needs to prevent long balls along the sline.

Using the proposed concepts, analyses like this one could be automated and computed automatically before a game. By doing so, dangerous opponent players can be easily identified and, together with video footage, dangerous episodes shown to the team. The system also proposes a way to decrease the dangerousness of these players by preventing the right passes, and also, these could be automatically retrieved from videos for a team briefing.

## 6. Conclusions

We incorporated data-driven movement models into measures of space and control that have been originally proposed by Fernandez and Bornn (2018). We highlighted differences between their original and our proposed approach and provided empirical evidence for the usefulness of our approach: using player movement models as the underlying influence of the player distinguished from by spatially clearly confined areas and significant correlations with quantifiable metrics such as xG. On this basis, we devised a novel space generation measure that allowed to credit generated space to either the pass giver or pass receiver. Both could play an important role when it comes to opponent analysis and analyzing games. As an example, we showed that the new measure can be used to automatically identify key players and to provide insights on how key passes to these players could be prevented.

## Data Availability Statement

The data analyzed in this study is subject to the following licenses/restrictions: Data is owned by the German league (DFL) and must not be disclosed. Requests to access these datasets should be directed to www.dfl.de.

## Author Contributions

All authors listed have made a substantial, direct and intellectual contribution to the work, and approved it for publication.

## Conflict of Interest

The authors declare that the research was conducted in the absence of any commercial or financial relationships that could be construed as a potential conflict of interest.

## Footnotes

^1^Fernandez and Bornn (2018) only provide a graph without any formula for the function they used in their study. We reproduced this function by capturing some coordinates from the plot and transformed these points into a feature matrix that contains 3-degree polynomial combinations for each data point. This matrix is learned using the ridge regression model (Hoerl and Kennard, 1970), and hyper-parameter selection is based on a leave-one-out cross-validation on the negative mean squared error.

^2^Alternatively, parametric approaches like adapting a Gaussian with maximum likelihood or a Gaussian mixture using expectation maximization could be pursued. However, the former cannot appropriately represent the multi-modal player distributions (cmp. Brefeld et al., 2019) and there remains the problem of choosing the number of mixing components in the latter. We simply circumnavigate these issues by staying non-parametric.

^3^The actual timestamp of the ball reception is difficult to determine due to noise in the data. In this study, we use a heuristic to select the point in time when the ball position is in a radius of 1.5 m around the receiving player. This heuristic is a trade-off between accuracy and the amount of successful passes that are actually detected in the tracking data.

^4^For simplicity, we choose a simple model with only a single feature. For higher predictive accuracies, we suggest to learn more sophisticated (possibly non-linear) functions to estimate the time horizons using additional features like actual player/ball positions and/or velocity vectors.

^5^The influence of the player also depends on the position of the ball **p**^*b*^. For notational simplicity in the notation, this is omitted and is implicitly included by the time *t* and the positions of all actors at that time.

^6^The proposed grid size trades-off accuracy and computation time. Other values are certainly possible.

^7^Comparing to traditional measures like the number of shots leads to similar outcomes with slightly lower correlations since data are more noisy.

^8^We use *xG* and *xA* values from https://fbref.com, provided by StatsBomb.

## A. Appendix

Last but not least, we study the impact of space generation on the market value of players. We use market values of the 2017/18 season as a quality indicator for the players gathered from an online platform^9^ that have been shown to correlate with actual transfer fees and even with the outcome of soccer tournaments (Franck and Nüesch, 2012; Bryson et al., 2013; Gerhards et al., 2014). We use a standard ordinary least squares (OLS) linear regression analysis to understand the relationship between market values (response variable) and space generation measurements (independent variables). Usually in soccer, teams within the same league have very different financial resources, and therefore, some teams can afford buying and paying players with higher market values than others. So, we factor in the team name as fixed effects into the model. As both response and independent variables are exponentially distributed, we need to log-transform them before fitting the models to meet the basic assumptions of the OLS model.

Table A1 summarizes the results. For the summed up passing and receiving space generation metrics *SG*_*total*_, the model coefficient suggests that market value of a player is 6.5% higher if the player is able to increase his performance in this category by 10%, i.e., the player with the median value of EUR 7.5 m would be worth almost EUR 8 m. We observe a similar effect when considering *SG*_*rec*_ alone. Here, a 10% increase in the receiver metric would account for a 3.4% increase in market value. For the passing metric *SG*_*pas*_, we find a significant relationship only for midfielders. Nevertheless, the influence this metric has on the market value is the highest as market value would be 9.3% higher if the *SG*_*pas*_ metric increases by 10%. This observation also matches with the traditional role for midfield players who usually need to have good play-making abilities. Although many other parameters are factored into the estimation of market value, the ability to create high-quality space as passer or pass receiver is something that is of great interest for soccer teams and clearly results in corresponding market values.

**Table A1:** Linear model results for relationship between market values and *SG* metrics.

**Formula**	**Coefficient**	***p*-value**
log(*market value*) ~ log(*SG*_*total*_) + *team*	0.65	0.013
log(*market value*) ~ log(*SG*_*rec*_) + *team*	0.35	0.029
log(*market value*) ~ log(*SG*_*pas*_) + *team* (only midfield players)	0.94	0.035
